# Effective management of lupus nephritis using a novel combination therapy with low-dose steroids: a case report

**DOI:** 10.1007/s40620-025-02361-y

**Published:** 2025-08-08

**Authors:** Niloufar Ebrahimi, Duvuru Geetha, Joselyn Reyes-Bahamonde, Craig W. Zuppan, Amir Abdipour, Sayna Norouzi

**Affiliations:** 1 Department of Medicine, Division of Nephrology, Loma Linda University Medical Center, Loma Linda, CA USA; 2 Division of Nephrology, Johns Hopkins University, Baltimore, MD USA; 3 Division of Nephrology, St. Luke’s University Health Network, Bethlehem, PA USA; 4 Division of Nephrology, Temple University, Philadelphia, PA USA; 5 Department of Pathology, Loma Linda University Medical Center, Loma Linda, CA USA

**Keywords:** Lupus nephritis, Belimumab, Voclosporin

## Abstract

Lupus nephritis (LN) is the predominant severe manifestation of systemic lupus erythematosus (SLE). The approach to managing LN is highly heterogeneous due to the clinical heterogeneity of the disease. Here, we report a 28-year-old Caucasian female with refractory biopsy-proven active LN class III and V, diagnosed  approximately six years prior to presentation who was successfully managed with both belimumab and voclosporin. Her laboratory findings were significant for high-titer anti-dsDNA antibodies (> 300), low complements, and increasing proteinuria despite ongoing treatment with MMF and low-dose prednisone. The patient fulfilled the EULAR/ACR classification criteria for SLE, achieving a score of 28, reflecting significant systemic disease involvement. The patient tolerated the combination of volcosporin and belimumab well for nine months. She did not report any specific side effects during this time and was in clinical remission with no signs of systemic flare-up based on the Systemic Lupus Erythematosus Disease Activity Index 2000 (SLEDAI-2K). This case highlights the potential of combining belimumab, voclosporin, mycophenolate mofetil (MMF), and low-dose steroids in selected patients.

**Graphical abstract:**

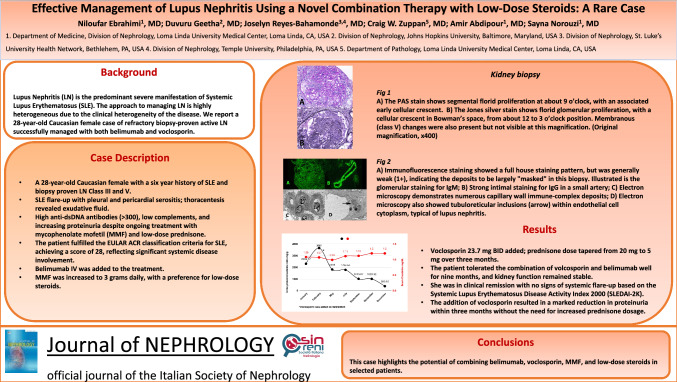

## Introduction

Lupus nephritis (LN) is present in approximately 50% of patients with systemic lupus erythematosus (SLE). However, it is associated with a higher risk in those of African ancestry and those positive for anti-Smith antibodies, and it predominantly impacts young women. It carries a potential risk for developing kidney failure [1, 2]. Active LN is a signal for initiating immunosuppressive treatment to prevent excessive urinary protein loss, nephron damage, and related complications [3]. Per KDIGO 2024 guidelines, active LN is defined as the presence of the following components in a kidney biopsy report, including endocapillary hypercellularity, neutrophils and karyorrhexis, fibrinoid necrosis, hyaline deposits, cellular/fibro-cellular crescents, and interstitial inflammation, each of which gets 0–3 scores, determining the activity [4]. In the recently published KDIGO guidelines on LN management, voclosporin with mycophenolic acid analogs or belimumab with mycophenolic acid analogs or low dose intravenous cyclophosphamide is now recommended for treating LN in the setting of active Class III/IV ± V LN, along with low dose steroids [4]. Moreover, according to the 2023 EULAR guidelines, adding either belimumab or voclosporin to conventional treatments such as mycophenolate mofetil (MMF) or cyclophosphamide is suggested. This approach aims to enhance the likelihood of achieving remission and mitigate the progression of kidney damage, thereby improving long-term outcomes for patients with LN [5]. The BLISS-LN study demonstrated that adding belimumab to the standard therapy of patients with proliferative LN significantly improved kidney response rates compared to placebo. This highlights the efficacy of belimumab in controlling disease activity in these forms of LN. In contrast, the AURORA-1 trial effectively managed membranous LN (class V) by adding voclosporin in combination with MMF and low-dose steroids. These findings suggest a therapeutic approach, with belimumab being more suitable for proliferative LN, while voclosporin is a better option for membranous LN [6, 7].

Herein, we report a case of refractory active LN, which was treated successfully with a combination of belimumab, voclosporin, MMF, and low dose prednisone.

## Case presentation

**Fig. 1:**
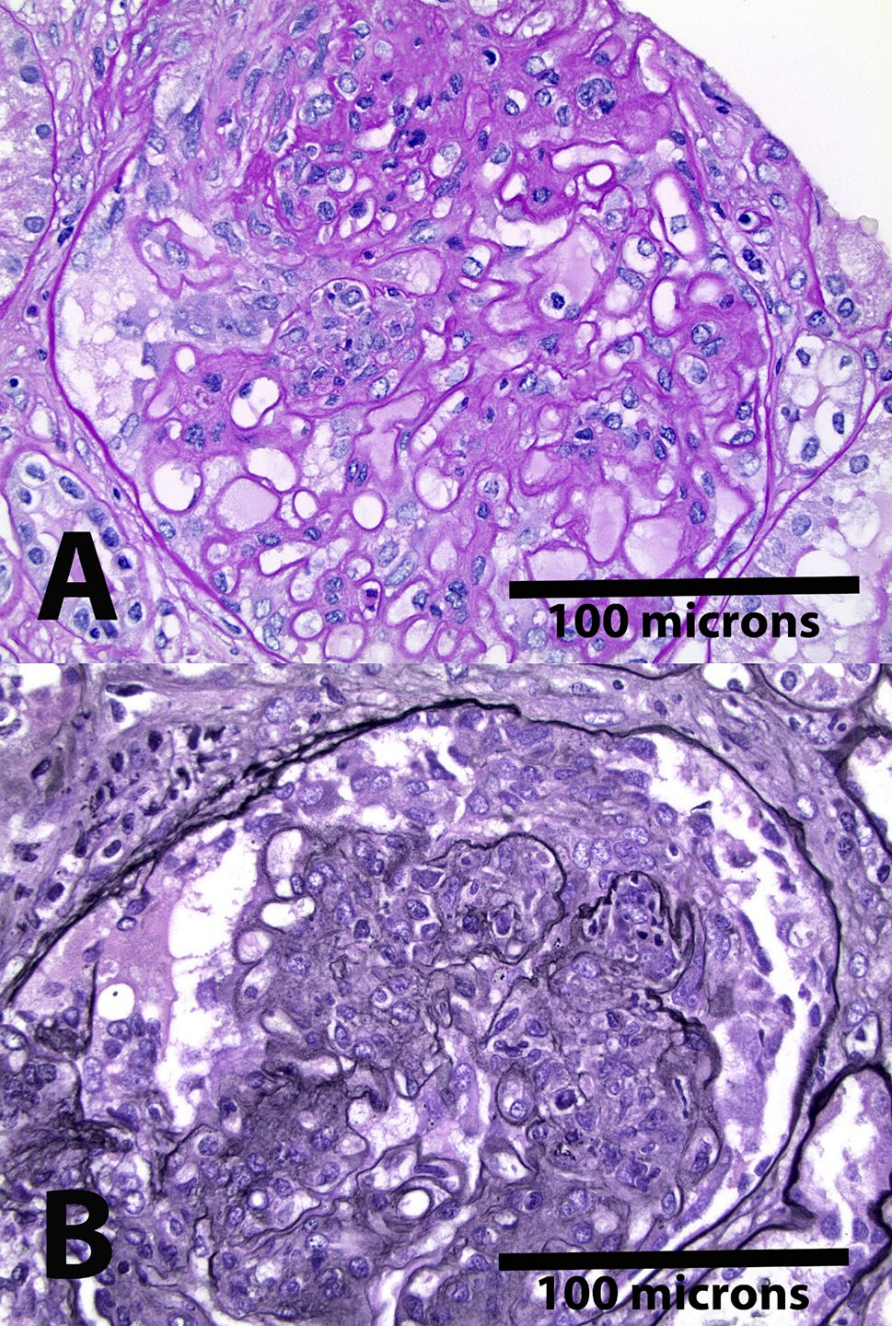
The kidney biopsy revealed focal active lupus nephritis. **A** The Periodic acid-Schiff (PAS) stain showed segmental florid proliferation at about 9 o’clock, with an associated early cellular crescent. **B** The Jones silver stain showed florid glomerular proliferation, with a cellular crescent in Bowman’s space, from about 12 to 3 o’clock position. Membranous (class V) changes were also present but not visible at this magnification. (Original magnification, × 400)

**Fig. 2:**
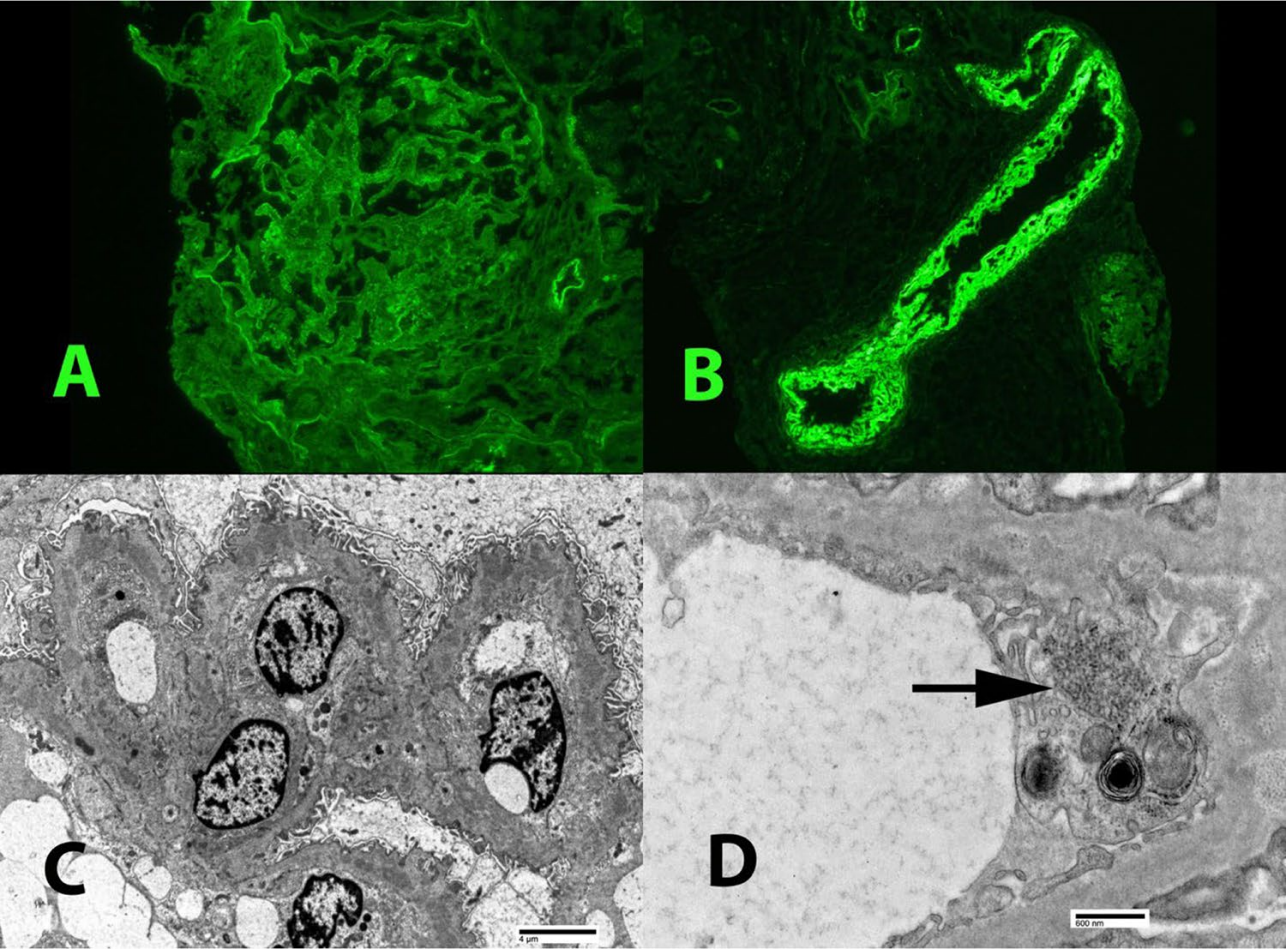
**A** Immunofluorescence staining showed a full house staining pattern, but was generally weak (1 +), indicating the deposits to be largely “masked” in this biopsy. Illustrated is the glomerular staining for IgM; **B** Strong intimal staining for IgG in a small artery; **C** Electron microscopy demonstrated numerous capillary wall immune-complex deposits; **D** Electron microscopy also showed tubuloreticular inclusions (arrow) within endothelial cell cytoplasm, typical of lupus nephritis

**Fig. 3:**
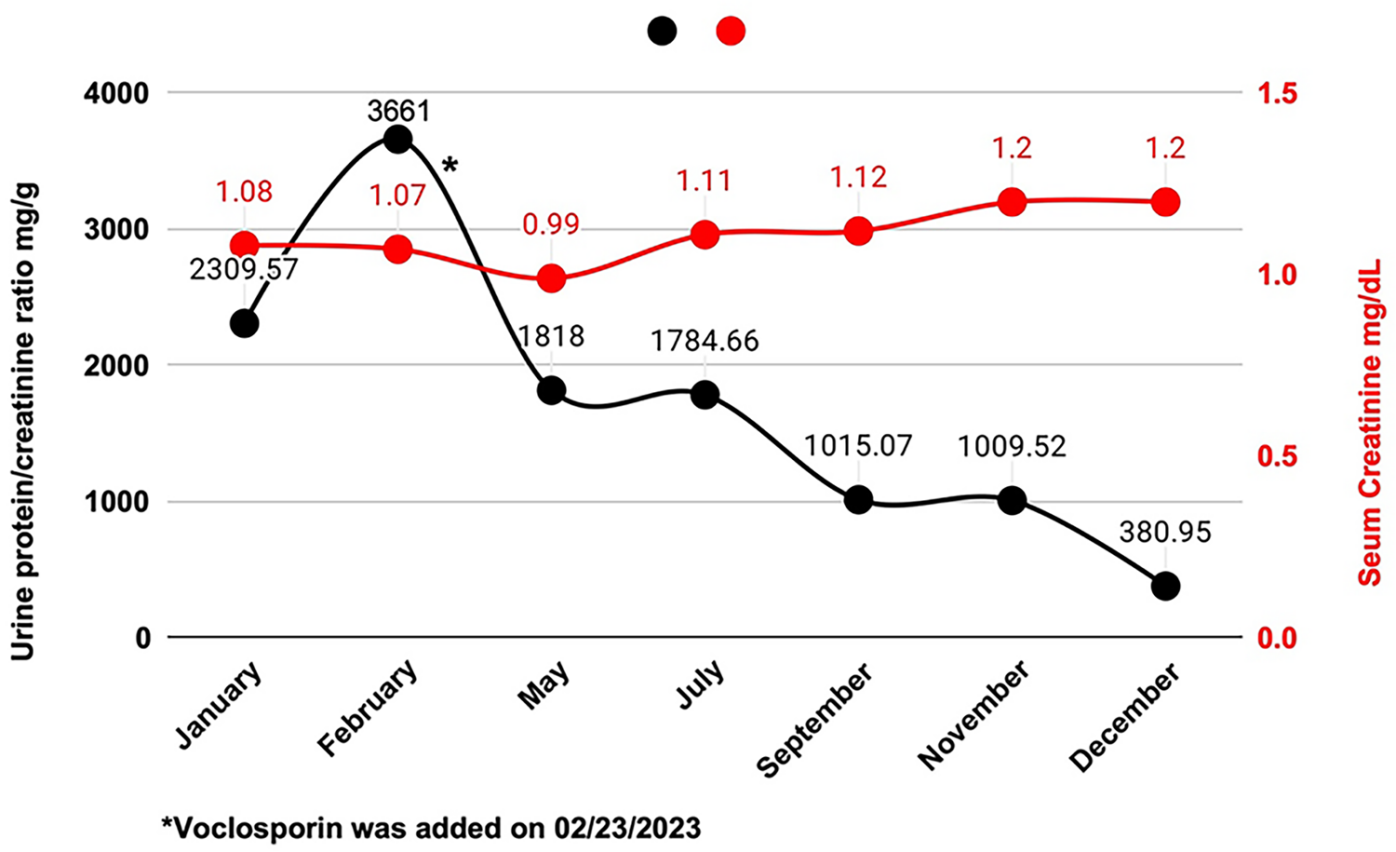
This figure exhibits the trend of urine protein/creatinine ratio (UPCR) changes along with serum creatinine levels before and after adding voclosporin to the patient's therapeutic regimen

A 28-year-old Caucasian female, diagnosed with SLE and LN class III and V approximately six years prior to presentation, presented with an SLE flare-up, which had occurred mostly twice a year due to medication noncompliance. Each flare-up was managed with pulse steroids and high-dose MMF. Her current extra-renal manifestations included pleural and pericardial serositis, with thoracentesis revealing exudative fluid. Her laboratory findings were significant for high-titer anti-dsDNA antibodies (> 300), low complements, and increasing proteinuria despite ongoing treatment with MMF and low-dose prednisone. The patient fulfilled the EULAR/ACR classification criteria for SLE, achieving a score of 28, reflecting significant systemic disease involvement [8]. After a multidisciplinary discussion, and considering the patient’s preference for not taking high dose steroids, her history of obstructive uropathy due to hydronephrosis of the left kidney resulting in nephrostomy tube placement, and recurrent urinary tract infections, belimumab was added to her treatment; starting with IV then switched to SC later on. Throughout the treatment, the dosage of MMF was increased to 3 g daily. After more than three months of treatment with the above regimen, a subsequent kidney biopsy was done in August 2022. The kidney biopsy revealed focal proliferative and membranous lupus nephritis, Class III + V with moderate activity, including focal necrotizing and crescentic lesions, and mild to moderate chronicity along with moderate arterio- and arteriolosclerosis (Fig. 1). As the biopsy result showed persistent active lupus nephritis, laboratory results indicated no significant improvement in proteinuria. Immunofluorescence demonstrated a full house pattern of capillary wall staining. Electron microscopy showed subepithelial, intramembranous, and mesangial immune deposits, along with segmental subendothelial deposits. Additionally, there was approximately 50–60% podocyte foot process effacement (Fig. 2). After a detailed discussion with the patient, who expressed a strong preference for avoiding high-dose prednisone, voclosporin was introduced to her existing medication regimen. One month after starting voclosporin 23.7 mg BID, her prednisone dosage was reduced from 20 to 15 mg daily, and it was further tapered down to 5 mg daily within two weeks after three months of voclosporin therapy. The addition of voclosporin resulted in a marked reduction in proteinuria within three months without the need for increased prednisone dosage (Fig. 3). The patient tolerated the combination of volcosporin and belimumab well for nine months. She did not report any specific side effects during this time and was in clinical remission with no signs of systemic flare-up based on the Systemic Lupus Erythematosus Disease Activity Index 2000 (SLEDAI-2K) (None- 0 score for extrarenal, total score 4 for proteinuria, which was improving, and low complement levels, which were significantly improved) (Table 1) [9].

**Table 1:** Patient’s laboratory results one month before adding belimumab, 3 months after adding belimumab, and 3 months after adding voclosporin to her therapeutic regimen

Lab parameter	On relapse	9 months after initiating belimumab	3 months after initiating voclosporin	Reference range
Creatinine	0.8	1.07	1.1	0.7–1.3 mg/dL
eGFR	93	73	71	> 59 ml/min/1.73
UPCR	7.6	3.6	1.8	g
C3	55.6	84.7	85	70–170 mg/dL
C4	7.4	10.7	9	11–40 mg/dL
dsDNA	> 300	14	9	1–4 IU/mL

*eGFR* estimated glomerular filtration rate, *UPCR* urine protein/creatinine ratio, *dsDNA* double-stranded DNA

## Discussion

Despite intensive treatment, around 60% of individuals affected by LN fail to achieve complete remission, leading to unfavorable long-term outcomes and complications such as chronic kidney disease (CKD) and progression to kidney failure at a young age [6]. Class III and Class IV LN are severe presentations of SLE which can result in permanent nephron loss if not promptly addressed with effective therapeutic regimens [4].

Belimumab functions by inhibiting B-lymphocyte stimulators, thus reducing B-cell survival and differentiation. This helps decrease the production of autoantibodies in LN. On the other hand, voclosporin, a calcineurin inhibitor, blocks T-cell activation by inhibiting calcineurin. This leads to a reduced immune response and stabilization of podocytes, which helps control proteinuria in membranous LN [10].

Combining voclosporin with mycophenolic acid analogs and low dose steroids in triple immunosuppressive regimens could be particularly beneficial for patients with refractory disease who experience high-grade proteinuria linked to extensive podocyte injury [4].

The BLISS study reported a more significant reduction in proteinuria by week 104 in patients treated with belimumab than placebo (43% vs. 32%, *P* = 0.03). Interestingly, the AURORA trial reported a reduction of proteinuria in week 52 in the voclosporin arm compared to the placebo. The rate of complete renal response was 30% in the BLISS-LN study in week 104 vs. 41% in the AURORA trial in week 52 [6, 7].

Additionally, belimumab is considered an adjunctive therapeutic approach and has recently received approval for treating LN based on the findings of a significant trial, the BLISS LN study [6, 11]. The patients in the BLISS LN study who were administered belimumab experienced a notably reduced risk of kidney-related events or death throughout the trial. Nevertheless, the response rate was characterized by a slow pace [6]. According to the AURORA trial, combining voclosporin with MMF and low-dose steroids resulted in a clinically and statistically superior complete kidney response rate compared to MMF and low-dose steroids alone while maintaining a comparable safety profile [7]. Four cases have been managed with simultaneous administration of belimumab and voclosporin, as Baum et al. reported, indicating improved or resolved proteinuria [12]. Significantly, in both the BLISS LN and AURORA trials, a proportion of patients failed to attain a complete therapeutic response, emphasizing the necessity for alternative approaches in treating LN [6, 7].

The BLISS LN study demonstrated enhanced results when belimumab was added to the treatment of patients with LN [6]. Furthermore, proteinuria exhibited a swift reduction within two weeks in the voclosporin AURORA trial [7].

Our case highlights the significant positive response achieved through the combination of belimumab and voclosporin, along with low-dose steroids, without any notable adverse effects. From our perspective, patients exhibiting a mixed pattern of proliferative and membranous LN on kidney biopsy represent an ideal profile for management with a combination of belimumab and voclosporin. Further research is warranted to assess the safety and efficacy of combining voclosporin and belimumab in combination with MMF and low dose, short term systemic steroids in managing LN.

## Data Availability

All data are included in this article. Further inquiries can be directed to the corresponding author.
