# Correction: Effective management of lupus nephritis using a novel combination therapy with low-dose steroids: a case report

**DOI:** 10.1007/s40620-025-02408-0

**Published:** 2025-08-26

**Authors:** Niloufar Ebrahimi, Duvuru Geetha, Joselyn Reyes-Bahamonde, Craig W. Zuppan, Amir Abdipour, Sayna Norouzi

**Affiliations:** 1 Department of Medicine, Division of Nephrology, Loma Linda University Medical Center, Loma Linda, CA USA; 2 Division of Nephrology, Johns Hopkins University, Baltimore, MD USA; 3 Division of Nephrology, St. Luke’s University Health Network, Bethlehem, PA USA; 4 Division of Nephrology, Temple University, Philadelphia, PA USA; 5 Department of Pathology, Loma Linda University Medical Center, Loma Linda, CA USA

**Correction: Journal of Nephrology** 10.1007/s40620-025-02361-y.

In this article, the figure 1 was incorrectly published with abstract figure. The correct figure 1 is given below.
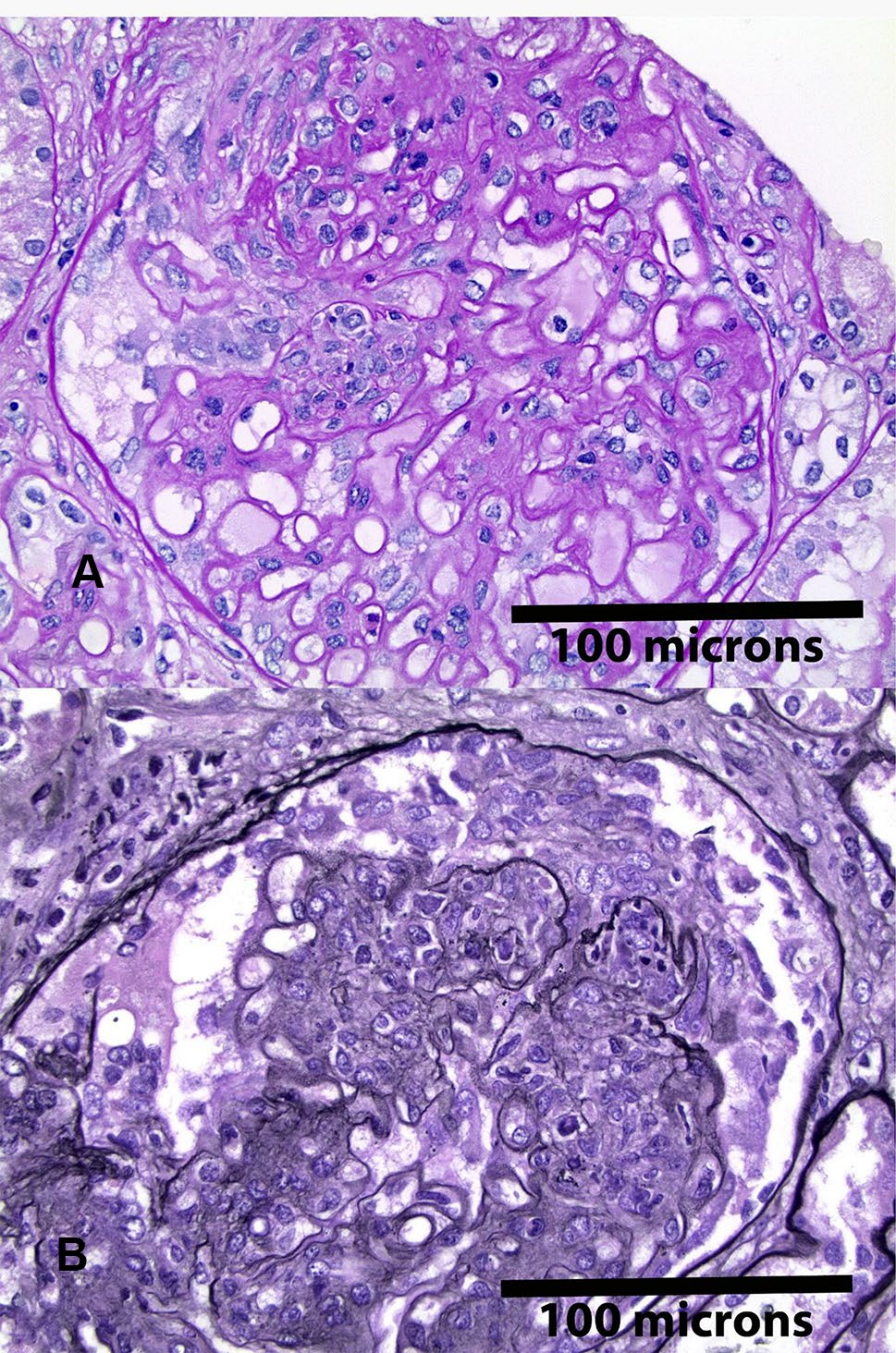


The original article has been corrected.

